# The Efficacy of Disinfection on Modified Vaccinia Ankara and African Swine Fever Virus in Various Forest Soil Types

**DOI:** 10.3390/v13112173

**Published:** 2021-10-28

**Authors:** Franziska Tanneberger, Ahmed Abd El Wahed, Melina Fischer, Sandra Blome, Uwe Truyen

**Affiliations:** 1 Institute of Animal Hygiene and Veterinary Public Health, Faculty of Veterinary Medicine, University of Leipzig, An den Tierkliniken 1, D-04103 Leipzig, Germany; Franziska.Tanneberger@web.de (F.T.); ahmed.abd_el_wahed@uni-leipzig.de (A.A.E.W.); 2 Institute of Diagnostic Virology, Friedrich-Loeffler-Institut, Suedufer 10, D-17493 Greifswald-Insel Riems, Germany; Melina.Fischer@fli.de (M.F.); Sandra.Blome@fli.de (S.B.)

**Keywords:** African swine fever virus, modified vaccinia Ankara virus, soil, disinfectant

## Abstract

African swine fever (ASF) has become a global threat to the pig industry and wild suids. Within Europe, including Germany, affected wild boar populations play a major role. Fencing and carcass removal in combination with the reduction in environmental contamination are key to control further spread. The handling of the ASF virus (ASFV) is restricted to high-containment conditions in Germany. According to the regulation of the German Veterinarian Society (DVG), modified vaccinia Ankara virus (MVAV) is the virus of choice to determine the efficacy of disinfection for enveloped viruses. The aim of this study was to use the MVAV as a guide to select the best possible disinfectant solution and concentration for the inactivation of ASFV in soil. Both viruses were tested simultaneously. In this study, two layers (top and mineral soil) of soil types from six different locations in Saxony, Germany, were collected. The tenacity of ASFV and MVAV were tested at various time points (0.5 to 72 h). The capabilities of different concentrations of peracetic acid and citric acid (approx. 0.1 to 2%) to inactivate the viruses in the selected soil types with spiked high protein load were examined under appropriate containment conditions. Around 2–3 Log_10_ (TCID_50_) levels of reduction in the infectivity of both ASFV and MVAV were observed in all soil types starting after two hours. For MVAV, a 4 Log_10_ loss was recorded after 72 h. A total of 0.1% of peracetic acid (5 L/m^2^) was sufficient to inactivate the viruses. A 4 log_10_ reduction in the infectivity of MVAV was noticed by applying 1% citric acid, while a 2 log_10_ decline was recorded with ASFV. In conclusion, comparing MVAV to ASFV for efficacy screening of disinfectant solutions has revealed many similarities. Peracetic acid reduced the infectivity of both viruses independently of the soil type and the existence of a high organic soiling.

## 1. Introduction

Over the last decade, the hemorrhagic contagious disease ASF has changed from an exotic disease of Sub-Saharan Africa to a considerable and tangible threat to the pig industry in Central Europe and Asia. The causative agent is a large, enveloped virus (175–215 nm) containing a genome of double-stranded DNA of 170–194 Kbp in length and belongs to the *Asfarviridae* family [1]. With its reintroduction into the European Union in 2014, the disease has also apparently found a fertile breeding ground in the abundant wild boar population. Based on previous experience on the Iberian Peninsula and Sardinia, wild boars were thus far not considered as a major and long-term reservoir for ASFV [2,3], and self-sustaining cycles were not anticipated in the beginning [4]. However, disease dynamics were completely different under the Northeastern European conditions and long-lasting endemic cycles without domestic pig involvement were established in all the affected countries once the virus was introduced into the wild boar population. Within the European Union and neighboring countries, wild boar populations in Bulgaria, Germany, Estonia, Latvia, Lithuania, Poland, Romania, Serbia, Slovakia, Ukraine and Hungary are currently affected [5].

Despite the high virulence of the viruses and considerable mortality among wild boar, these cycles have been self-sustained over several years now. In the absence of an oral vaccine or other direct means of therapy, control has to rely on hygiene measures including fencing, the removal and safe disposal of carcasses and a reduction in the susceptible population in adjacent areas [6,7]. Given the high tenacity of the virus in the environment [8,9], decontamination of the deathbed (soil/ground beneath the carcass) has been discussed. Soils in wild boar habitats have often a rather acidic pH that could lead to inactivation of ASFV, as was demonstrated by Carlson et al. [10]. However, decontamination is still recommended, and disinfection is complex as soil encompasses many organic contents, which influence the potency of disinfectants.

The most commonly used disinfectants are based on peracetic acid, formic acid, sodium hydroxide, citric acid or slaked lime/quick lime [11]. The concentration and route of application depend on the target surface, the ambient temperature and chemical properties (e.g., pH) [12,13].

In Germany, only commercial chemical disinfectants that are efficacy-tested according to German Veterinary Society (DVG) guidelines can be used for official disinfection procedures. The biosafety level of ASFV hampers extensive studies. According to the DVG protocols, MVAV is used as a test virus for efficacy testing as a representative of the enveloped viruses [14].

In this study, in a BSL-2 laboratory, the tenacity of MVAV and the inactivation with various concentrations of peracetic acid and citric acid were examined in two layers (topsoil, TS; and mineral soil, MS) of six soil types collected in the state of Saxony, Germany. The outcomes of this experiment were validated under high containment conditions using ASFV. The experimental setup is depicted in Table 1.

## 2. Materials and Methods

### 2.1. Soil Types

Various soil types were collected from different regions in Saxony, Germany (Table 2). The sampling and characterization of soil types and quality were performed under the supervision of the Sachsenforst, Pirna, Germany. For each soil, TS and lower sandy layer MS were collected. The soil samples were stored in airtight plastic bags and/or buckets at 10 °C. Freedom of ASFV and MVAV was confirmed using a quantitative polymerase chain reaction (qPCR, see below) for all soil samples. In addition, at the time of collection (April 2020), Germany was listed free of the virus by the (World Organization for Animal Health) OIE.

### 2.2. Viruses and Cell Cultures

MVAV was propagated in chicken embryo fibroblasts to obtain a titer of approximately 10^8^ TCID_50_/mL or higher. The cultivation was conducted at 37 °C and 5% CO_2_ in Dulbecco’s minimum essential medium (DMEM) supplemented with 10% fetal calf serum (FCS, PAA Laboratories, Pasching, Austria), 1% l-glutamine, 1% non-essential amino acids, penicillin (100 IU/mL; Biochrom, Berlin, Germany) and streptomycin sulphate (100 lg/mL; Biochrom). Virus titration was performed by microscopically assessing the virus-specific for cytopathogenic effects (CPE) for up to 8 days to determine the 50% tissue culture infectious doses (TCID_50_) according to the Spearman–Kaerber method.

A tissue culture adapted ASFV strain originating from the ASFV strain “Estonia 2014” [15] (virus passage 15) with a titer of 10^6.75^ TCID_50_/mL was used for ASFV proof-of-concept studies. The ASFV-permissive wild boar lung cell line WSL-R-HP (CCLV-RIE 1346, FLI Insel Riems, Greifswald, Germany) was used for virus propagation (DMEM (E, high-glucose, 4.5 g/L)). The TCID_50_ was determined by indirect immunofluorescence after 3 to 4 days using a commercial p72 specific antibody (Ingenasa, Madrid, Spain) and an Alexa 488 conjugate goat anti-mouse IgG (H + L) (Fisher Scientific, Schwerte, Germany).

### 2.3. Tenacity of the Viruses

To examine the stability of MVAV and ASFV in soil, the viruses were dried on stainless steel germ carriers and buried at 0.5 and 5 cm in the TS and MS of the six soils. Briefly, the MVAV or ASFV was mixed with FCS (PAA Laboratories, Pasching, Austria) to obtain a protein load of 40% in the mixture. The mixture was spread on the sterile steel germ carriers (50 µL for 10^8^ TCID_50_/mL MVAV and 250 µL for 10^6^ TCID_50_/mL ASFV) and air dried under the laminar flow at 20–22 °C for 30–60 min. Thereafter, the virus-germ carrier was buried at 0.5 and 5 cm under the soil in a sterile glass beaker (diameter of 5 cm). The beakers were incubated at 10 °C for 0.5, 1, 2, 24 and 72 h for MVAV, and 2, 24 and 72 h for ASFV. To recover the remaining viruses, the germ carrier was subsequently washed 15 times with 5 mL of chilled PBS for MVAV carriers and 2.5 mL for ASFV carriers. The collected fluid was filtered through a 0.45-micrometer syringe filter. A ten-fold serial dilution of the filtrate was prepared and 100 µL was used for determining the TCID_50_. In addition, DNA was extracted from the samples and examined using a qPCR. The stability of the viruses at various time points in each soil type and layer were examined at least 9 times for MVAV and 3 times for ASFV. The TCID_50_ values were subtracted from the control, which was represented by a germ carrier dried without contact with soil.

### 2.4. Disinfection in Soil

The following two commercially available chemical disinfectant substances were examined that are recommended by the DVG for use in animal diseases: peracetic acid (15% stock solution, pure, AppliChem, Darmstadt, Germany) and citric acid (192.13 g/mol, pure, anhydrous, Carl Roth, Karlsruhe, Germany). Both disinfectants were diluted to obtain concentrations of 2, 1, 0.5 and 0.1%. In addition, 0.01% and 0.001% peracetic acid were used. To achieve the maximum performance of the disinfectant, the dilutions were prepared freshly 15 min before the start of the experiment. In contrast to the tenacity experiment, screening of the disinfectants was carried out as a suspension to simulate the real case scenario. Briefly, a volume of 2.7 (width) + 1.5 (height) cm of TS and MS of each of the 6 soil types, corresponding to about 3 mL of soil, depending on the water content, were placed in 50-milliliter centrifuge tubes. Then, 3 mL of virus (10^8^ TCID_50_/mL for MVAV and 10^6.75^ TCID_50_/mL for ASFV) and 4 mL of FCS were added. After vortexing for 5 s, 2.9 mL of the disinfectant solution containing 3.4-fold the desired concentration was vortexed for 5 s. As an example, for a final working concentration of 1%, 3.4% of the disinfectant was used. The tube was incubated at 10 °C for 2 h. Thereafter, 2.1 mL of ice-cold PBS was added and mixed. The mix was sonicated in an ultrasound bath (Bandelin Sonorex Super RK 103 H, Berlin, Germany) at 4 °C for 5 min. To obtain the supernatant, the mixture was centrifuged at 4500 rpm and 4 °C for 5 min. Around 5 mL of the supernatant was filtrated (Filtropour 0.45 µm, Sarstedt, Nümbrecht, Germany). Ten-fold serial dilutions were prepared and added to cell culture to determine the loss of infectivity. Generally, 100 µL was applied to the cells in a 96-well plate. To avoid cell toxicity, in some cases, 25 µL was applied to either 24- or 96-well plates. At the same time, qPCR was conducted using the soil supernatant. The experiment for each concentration of the disinfectant solution in each soil type and layer were conducted in duplicates. In the control soil, PBS was used instead of the disinfectant solution.

### 2.5. Disinfectant Volume Tests

The German Federal Ministry of Food and Agriculture (BMEL) recommended 5 L/m^2^ of the final concentration of the disinfectant solution on surfaces [16]. In order to determine the penetrability of the disinfectants in various soil types, a germ carrier containing MVAV was buried at a 0.5- and 5-centimeter depth. Briefly, a 250-microliter mixture of 150 µL of 10^8^ TCID_50_ MVAV and 100 µL of FCS were pipetted onto the germ carrier and air dried under the laminar flow at room temperature for 60 min. The virus-germ carrier was buried 0.5 and 5 cm under the soil in sterile perforated plastic boxes (diameter of 12.5 + 10.5 + 7 cm). A total of 65 mL of 0.5% peracetic acid was added. In the control boxes, water with standardized hardness level (WSH) was used [17]. The boxes were incubated at 10 °C for 2 h. The germ carrier was removed and washed 15 times with 2.5 mL of chilled PBS, then filtrated through a 0.45-micrometer sterile filter. A tenfold serial dilution of the flow was prepared and 100 μL was used for determining the TCID_50_. In addition, qPCR was conducted. For each depth and soil type, four values were determined.

### 2.6. DNA Extraction and qPCR

Nucleic acids from 200 µL of supernatant of germ carrier or soil suspension were extracted using the DNeasy kit (Qiagen, Hilden, Germany) according to the manufacturer’s recommendations. MVAV qPCR was performed using a Multiplex PCR Master Mix (Qiagen, Hilden, Germany) and EvaGreen (Jena Bioscience, Jena, Germany) on an Mx3000 p real-time cycler (Agilent Technologies, Santa Clara, CA, USA). Synthetic molecular DNA Standard was produced by GeneArt (Invitrogen, Carlsbad, CA, USA, GenBank accession number MK314713.1, nucleotide 147538 to 148127) and a serial dilution of 10^5^–10^1^ was applied for DNA quantification. The reaction total volume was 25 µL, containing 1 µL of each MVAV primer (10 µM) published previously [18] and 0.1875 µL of EvaGreen. The temperature profile was a 15-minute initial activation at 95 °C, followed by 40 cycles of denaturation for 10 sec at 95 °C, annealing/extension for 20 s at 55 °C and elongation for 20 s at 72 °C, as recommended by the manufacturer.

In case of ASFV, nucleic acids from the samples were extracted on a KingFisher 96 flex platform (Thermo Fisher, Waltham, MA, USA) using the NucleoMag Vet kit (Macherey-Nagel, Düren, Germany) according to the manufacturer’s instructions. Nucleic acids were analyzed using qPCR (King et al., 2003) in combination with an internal control based on an EGFP detection system [19] on a Bio-Rad CFX real-time cycler (Bio-Rad Laboratories, Hercules, CA, USA). A genomic ASFV DNA standard was used for determining the genome copy numbers of the samples.

## 3. Results

### 3.1. MVAV and ASF Tenacity in Soil

**Figure 1:**
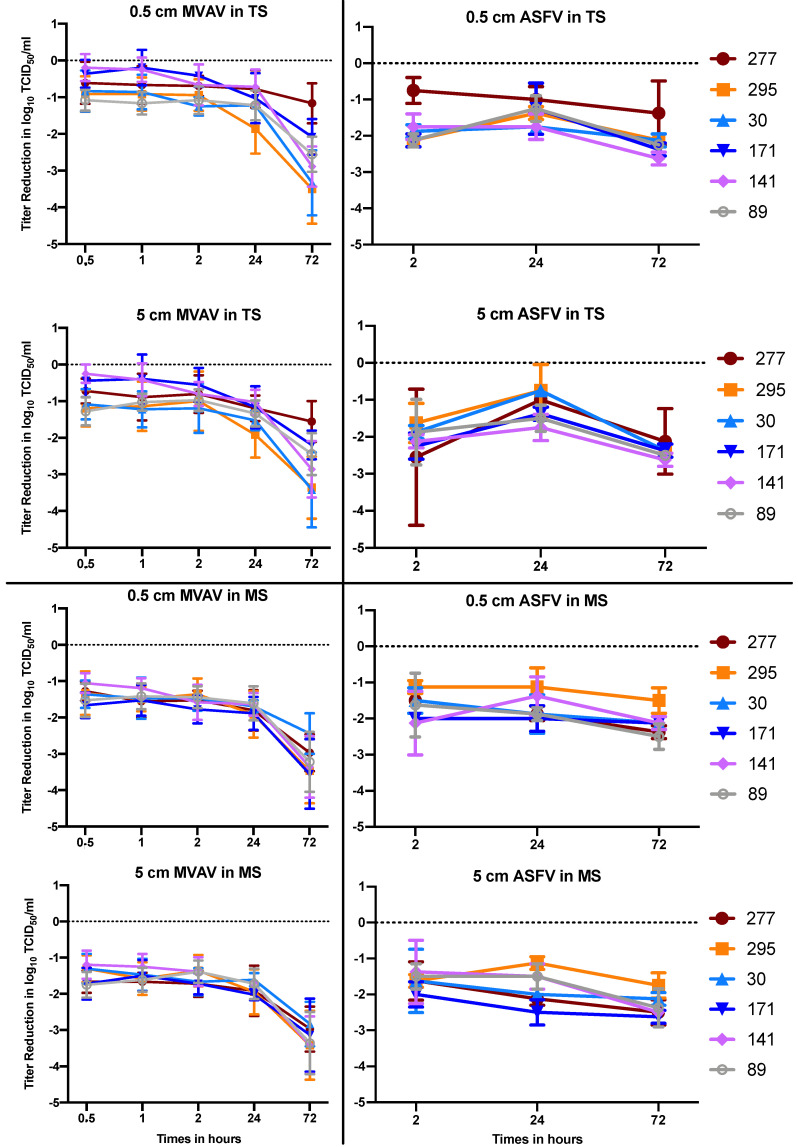
Comparison of the tenacity of MVA (**left**) and ASFV (**right**). Top 4 panels are topsoil (TS) and lower 4 panels are mineral soil (MS). The mean value and standard deviation of at least double measurements are shown for each test point.

The virus-covered germ carriers were buried in two different layers (0.5 and 5 cm) of six forest soil types from Saxony, Germany for up to 72 h. In general, a maximum titer reduction of ±2–3 log levels was recorded for MVAV and ASFV (Figure 1). While the MVAV tenacity was reduced dramatically after 72 h, ASFV was more stable. Despite the 0.5 log difference between the stabilities of the viruses in TS and MS, no other significant variation was recorded. This is also true for all the soil types. Interestingly, no differences between the viral load between the control and the buried germ carrier were recorded using qPCR, which indicates a true loss of infectivity rather than the removal of virus particles (Supplementary File S1).

### 3.2. MVAV and ASFV Inactivation in Soil

**Figure 2:**
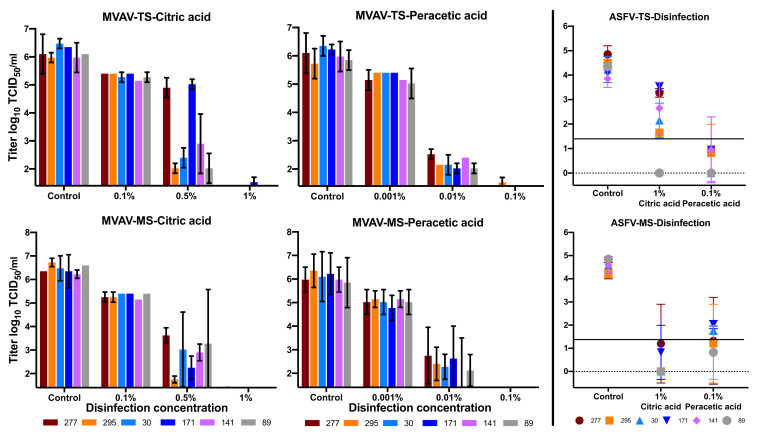
Comparison of the disinfection of MVAV and ASFV (limit of detection 10^1.4^ log_10_ TCID_50_/mL). Top 3 panels are topsoil (TS) and lower 3 panels are mineral soil (MS). The mean value and standard deviation of at least double measurements are shown for each test point. The virus titers were calculated in the case of the MVAV by CPE and in the case of the ASFV by immunofluorescence. Therefore, different evaluation schemes were used.

Various concentrations of disinfectant solutions were used to inactivate MVAV and ASFV in the presence of high organic soiling (40% protein load, FCS). For peracetic acid, 0.1% was sufficient to reduce the MVAV viral titer by four log levels, whereas 1.0% of citric acid was required to achieve the same reduction (Figure 2). The disinfectant solution behaved similarly in both TS and MS for MVAV. In contrary, for ASFV, 0.1% peracetic acid overperformed the 1.0% citric acid in both TS and MS (Figure 2). MVAV was inactivated in all types of soils without any significant differences, while variations were observed between the soil types with ASFV (Figure 2). The viral load as determined using qPCR for both the control and the experimental set revealed very close values (Supplementary File S1).

### 3.3. Penetration of the Disinfectants in Soil

**Figure 3:**
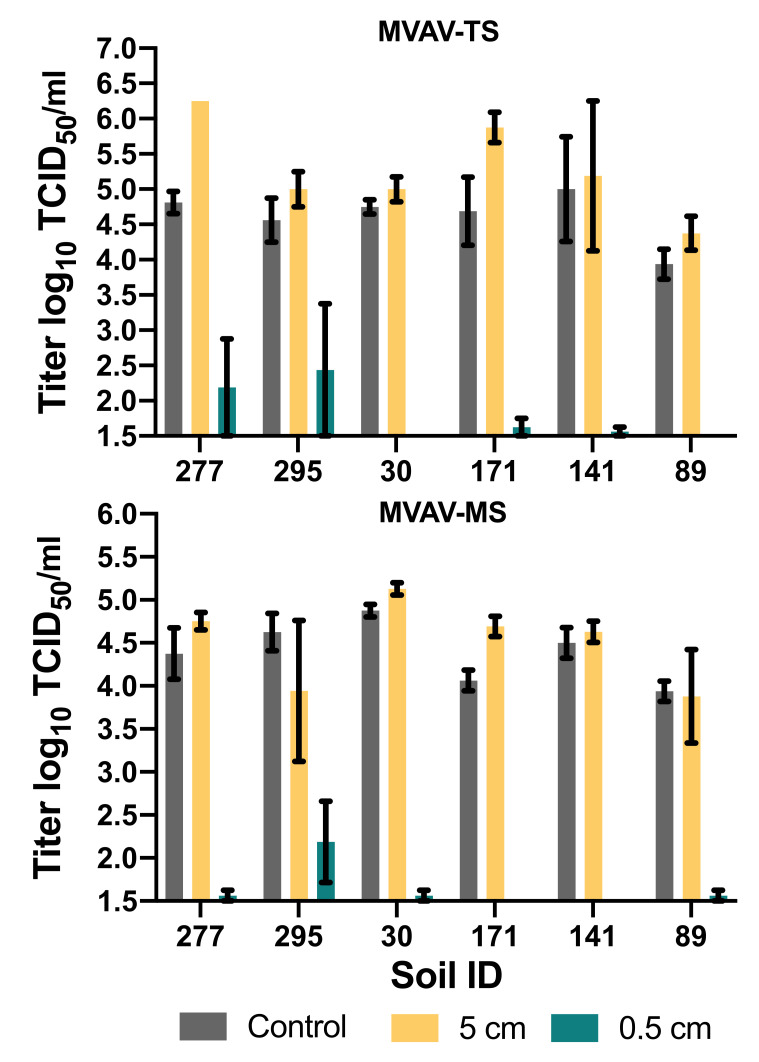
Penetrability of 0.1% peracetic acid on MVA-germ carriers at 0.5- and 5-centimeter depth in (**top panel**) in topsoil (TS), (**lower panel**) in mineral soil (MS); limit of detection 10^1.5^ log_10_ TCID_50_/mL.

Around 5 L/m^2^ of the disinfectants was sufficient to inactivate the viruses in the soil/MVAV/FCS suspension. The effectiveness of the disinfectant solution at various soil levels was examined by burying the MVAV germ carrier in 0.5 and 5 cm of TS and MS of each soil type. A sufficient reduction in the virus was only noticed at the 0.5-centimeter depth for all the soil except TS of 277 and 295 as well as MS of 295. At the 5-centimeter depth, no reduction was recorded. The TCID_50_ of both the control and the soil after disinfection remained the same for every soil (Figure 3).

## 4. Discussion

In the context of the current epidemic, several outbreaks started in wildlife with one of the most recent examples being Germany. Here, cases are currently observed in Brandenburg and Saxony [20]. The careful and complete disposal of carcasses of wild boar that succumbed to disease, as well as the removal or treatment of the contaminated soil under or around the carcass are necessary as recommended by the BMEL and the OIE [21,22]. An approach to minimize both the contact probability and movement of contaminated soil is the disinfection of the soil on site. Therefore, in a controlled experiment, we have screened the tenacity of both MVAV and ASFV in TS and MS of six different forest soils in Saxony, Germany. For MVAV and ASFV, a loss of titer by 2–3 log TCID_50_ levels can be observed after two hours independent of the soil type. Surprisingly, the effect of disinfection on MVAV and ASFV differed. To support the virus reducing effect of the forest soil (pH 4–6), our study started with the acidifying, environmentally friendly disinfectants peracetic and citric acid, commonly used during outbreaks of animal diseases. Peracetic acid accomplished around 4 log levels reduction in infectivity of both ASFV and MVAV in all the tested soil types, while only a limited effect was observed for citric acid for ASFV in TS. The mode of action of both disinfectants is very similar (destabilizing the lipid bilayer and denaturation of proteins; [23,24]). The tested soil types had pH values between 4 and 5.5, which have only a limited effect on the viruses (approx. pH 3 or lower will inactivate both ASFV and MVAV depending on protein load and time [23,25,26]). Peracetic acid is active at a wide range of pH values, while citric acid is more effective at a low pH (<3) [24], which may explain the better performance of peracetic acid in our study.

To simulate field conditions, a high protein load of 40% FCS was included and the experiment was performed at a temperature of 10 °C (required temperature for testing the efficiency of disinfectants by DVG [14], and close to the annual average temperature of soil in Saxony). The efficacy of citric acid was markedly decreased in the presence of high protein content or fluctuations in the ambient temperature [24]. The same is reported for peracetic acid [27] but was not observed in our experiment. Peracetic acid is well known for being less effective in the presence of protein contamination such as blood, feces or pus [27]. We tried to account for this fact in our experiments by adding a large amount of FCS (40%) to the virus inoculum, as it is important to keep in mind the more complex composition of other body excretes.

Selecting environmentally friendly disinfectants is crucial to minimize the pressure on the environment. Citric acid is an organic acid and peracetic acid dissociated to acetic acid, oxygen and water after a few hours [28]. Both have been extensively used in the food industry because of their low toxicity [29,30]. Therefore, both represent examples of eco-friendly substances, even after the application of large quantities.

Slight differences in the infectivity between the MVAV and ASFV were recorded. The reason may be the variation in the approaches that were used in determining the TCID_50_. In the case of MVAV, a microscopical examination of the cells for typical CPE, consisting of detached, enlarged and rounded cells, was conducted [31,32]. For ASFV, indirect immunofluorescence staining was applied.

Although various soil types were used in the experiment, no differences in virus stability or susceptibly to disinfectants were recognized. The only variations were seen with soils 277 (TS) and 295 (TS and MS), when the MVAV germ carrier was buried 0.5 cm deep in the soil (Figure 1). For the mentioned soil types, we observed a different penetration of the disinfectant due to the lower level of humidity. Therefore, a sufficient volume of disinfectant solution must be applied. The BMEL has previously recommended 5 L/m^2^ on surfaces [16], but this might not be applicable for all soil types. Although the 0.1% peracetic acid did inactivate the tested virus, we recommend using 0.5% peracetic acid to avoid the dilution effect of the excess humidity or soil water content or a potential loss of efficacy due to interactions with other substances. In other words, the humidity of the soil must be considered before applying any disinfectants.

MVAV was selected as a representative for enveloped viruses by DVG [33]. Both MVAV and ASFV have a complex structure including double envelopes and capsids. Historically, ASFV was assigned to the *Poxviridae* until 1998, when the *Asfarviridae* family was created [34]. The use of MVAV facilitated the conduction of the experiment under BSL-2 laboratory conditions and avoided higher containment requirements, which are legalized for handling ASFV in Germany.

## 5. Conclusions

This study demonstrates the similarities between MVAV and ASFV susceptibilities to peracetic acid in various forest soil types. More research is needed to screen other disinfectants as well as to investigate the inhibition effect of complex body fluids (e.g., blood) and other organic substances.

## Figures and Tables

**Table 1:** Experiment layout.

Virus	BSL	Part of Soil	Types of Soil	Depth for Tenacity	Tenacity Times	Disinfection (5 L/m^2^)
MVAV	BSL-2	topsoil; mineral soil	6	0.5 cm; 5 cm	0.5; 1; 2; 24; 72 h	0.1; 0.5; 1% citric acid 0.001; 0.01; 0.1% peracetic acid
ASFV	BSL-4	topsoil; mineral soil	6	0.5 cm; 5 cm	2; 24; 72 h	1% citric acid 0.1% peracetic acid

**Table 2:** List of the soil collected from different locations in Saxony, Germany.

ID *	Collection Site	Forest Stand	pH Topsoil	pH Mineral Soil
277	Oberholz/Leipzig Süd	deciduous forest, rich in conifers	5.32	5.53
295	Taura/Schöneiche	pine forest	4.03	3.92
30	Klitten/Bautzen	pine forest	4.01	4.32
171	Marienberg/Heinzebank (Erzgebirgskreis)	spruce forest	5.75	4.69
141	Bärenfels/Hetzdorf (Sächsische Schweiz/ Osterzgebirge)	spruce forest	4.24	3.91
89	Ottendorf	coniferous forest, rich in deciduous trees	4.01	4.32

* official names by the authority of Sachsenforst, Pirna, Germany.

## Data Availability

All data are available in the manuscript and the Supplementary Materials.

## Supplementary Materials

The following are available online at https://www.mdpi.com/article/10.3390/v13112173/s1, Supplementary File S1: contains the real-time PCR results.
