# Supplementary figures and images for: The Efficacy of Disinfection on Modified Vaccinia Ankara and African Swine Fever Virus in Various Forest Soil Types
Source: Viruses. 2021 Oct 28;13(11):2173. doi: 10.3390/v13112173 (PMC8618179; doi:10.3390/v13112173)

**Supplementary File S1. Containing all qPCR data:**

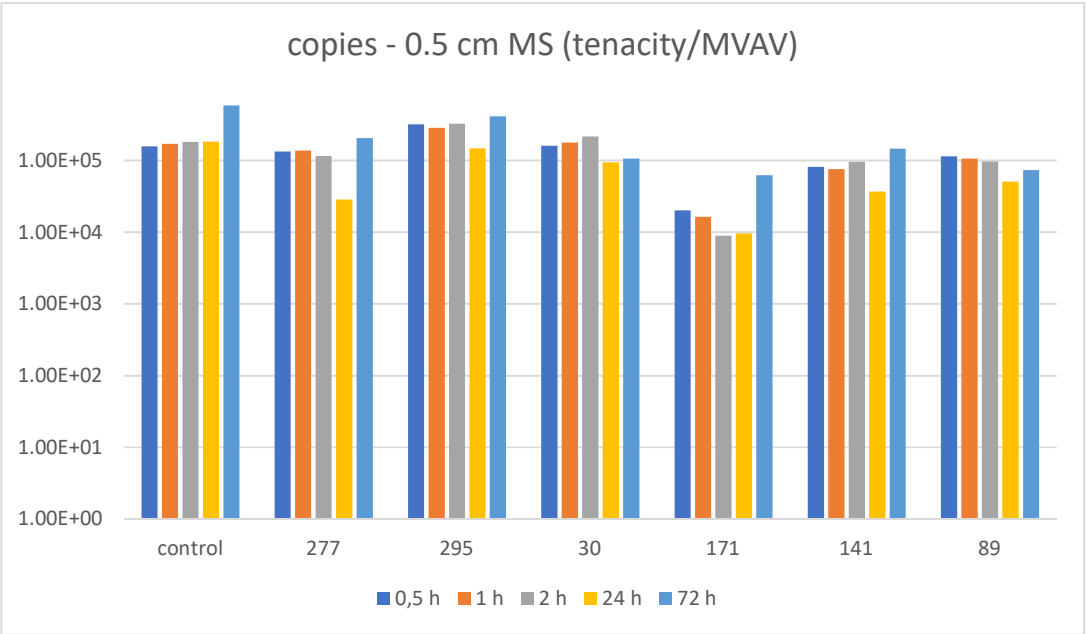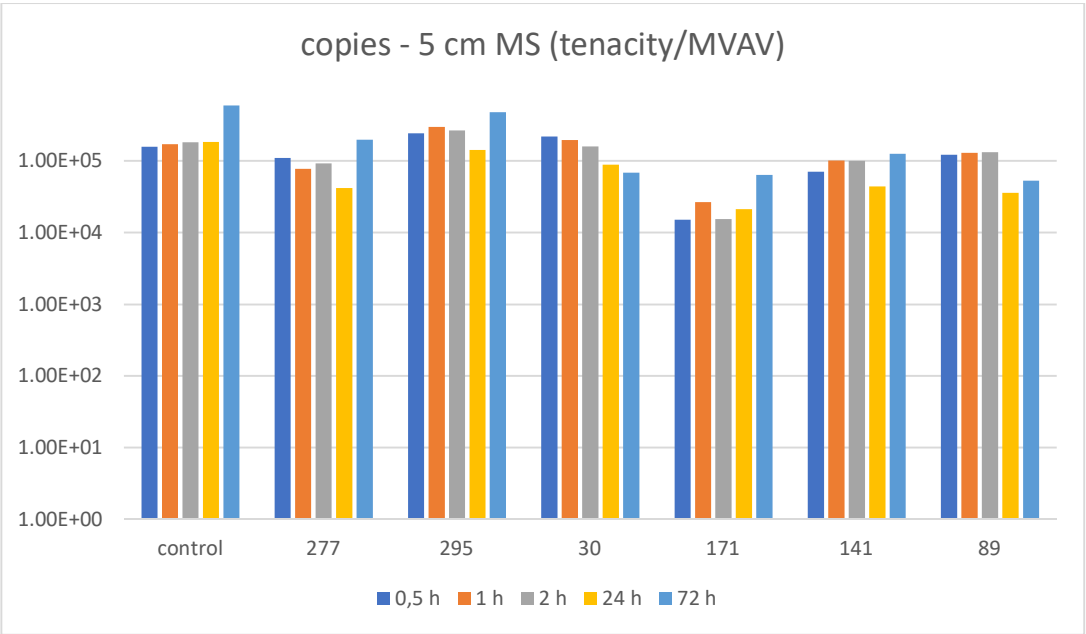

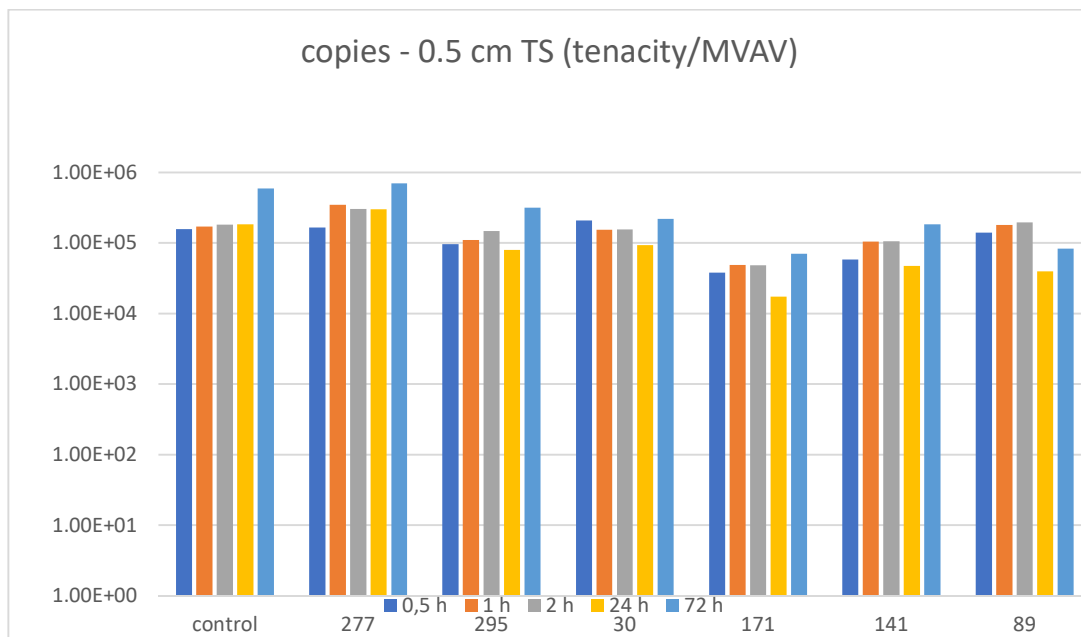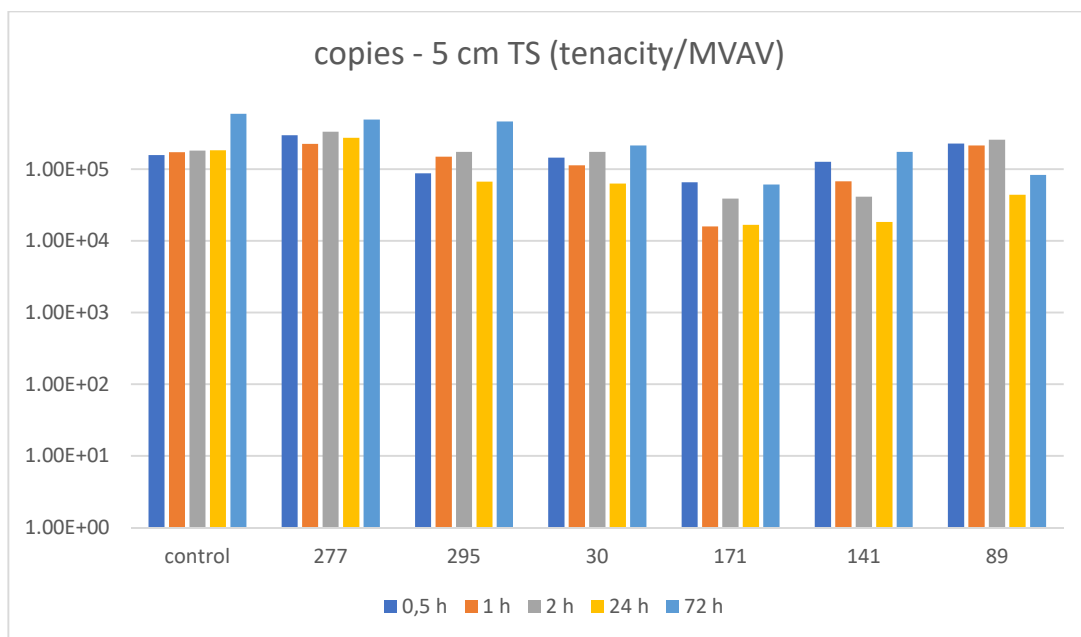

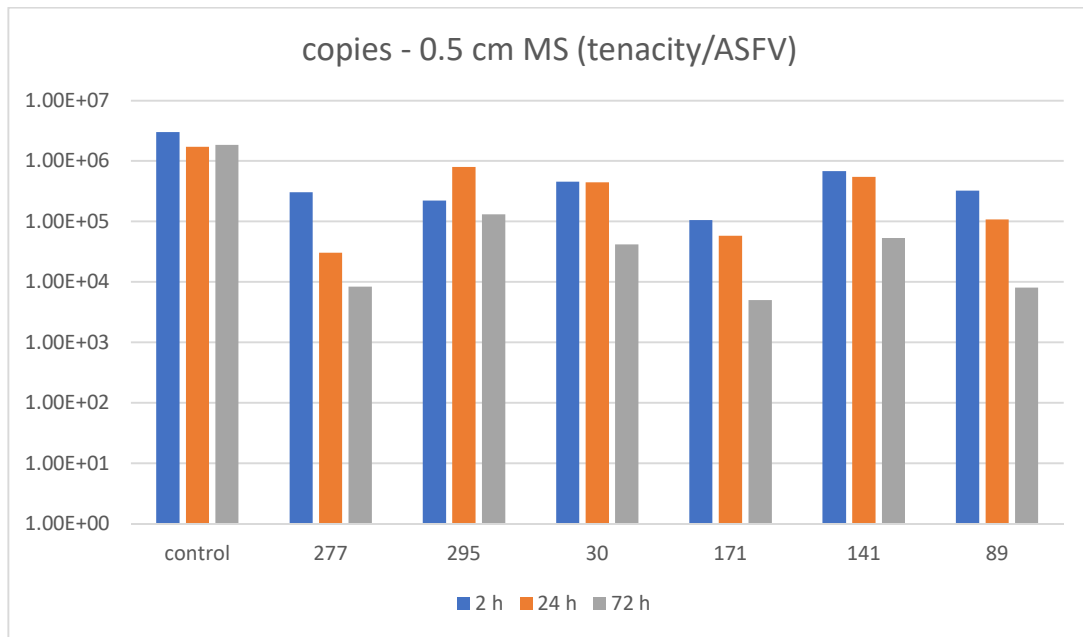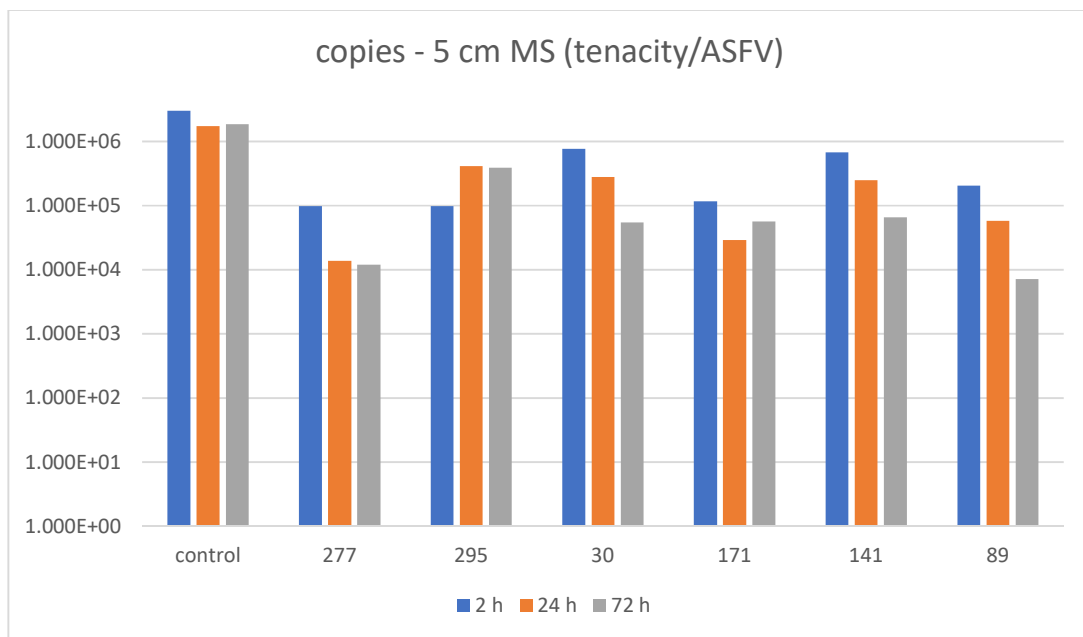

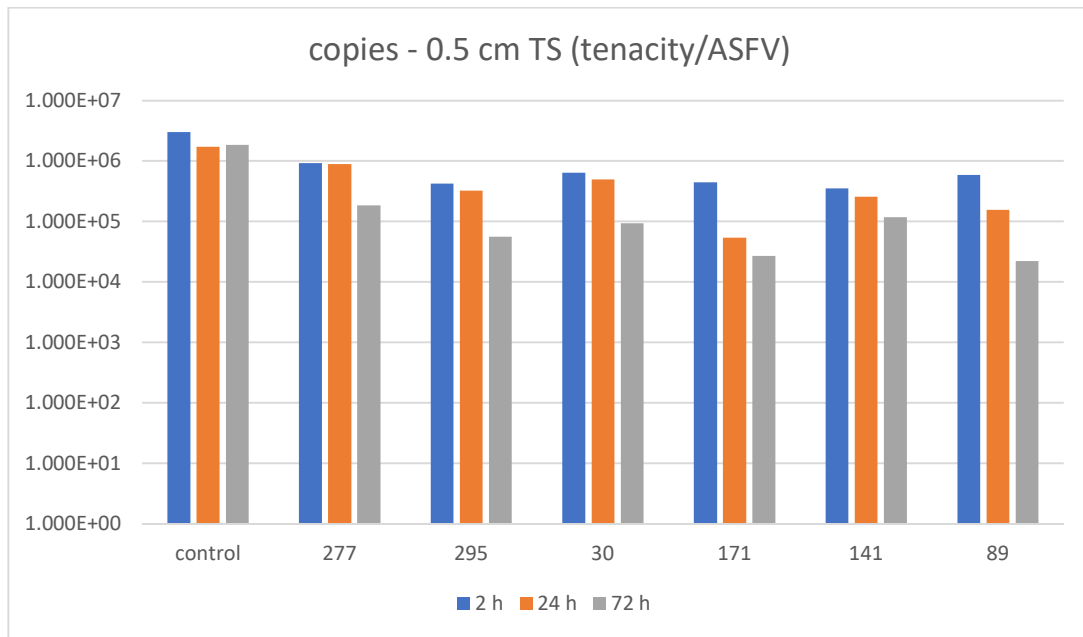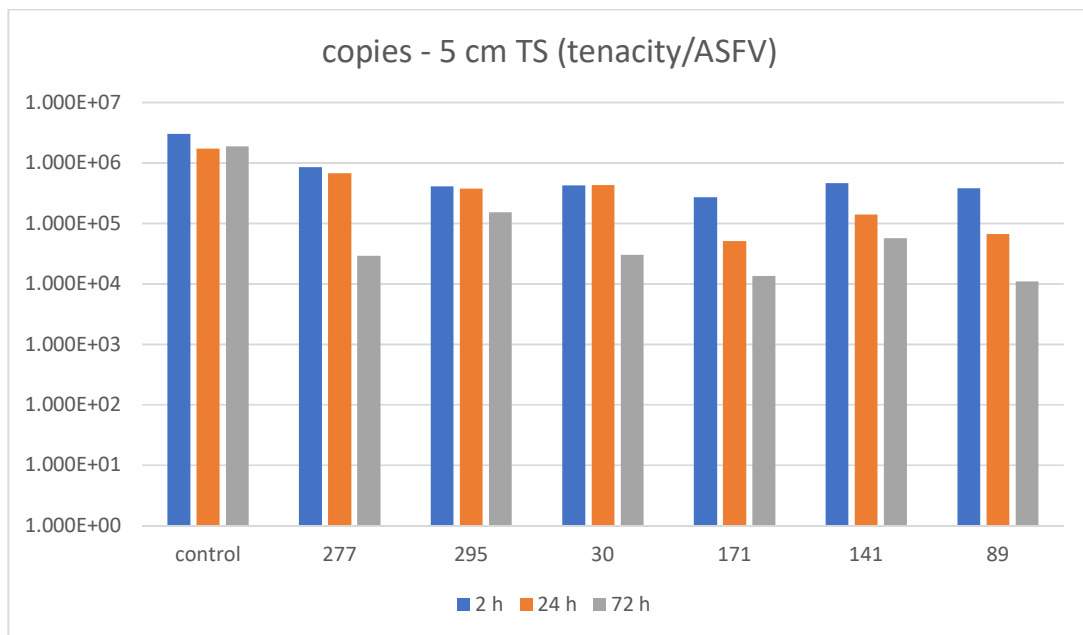

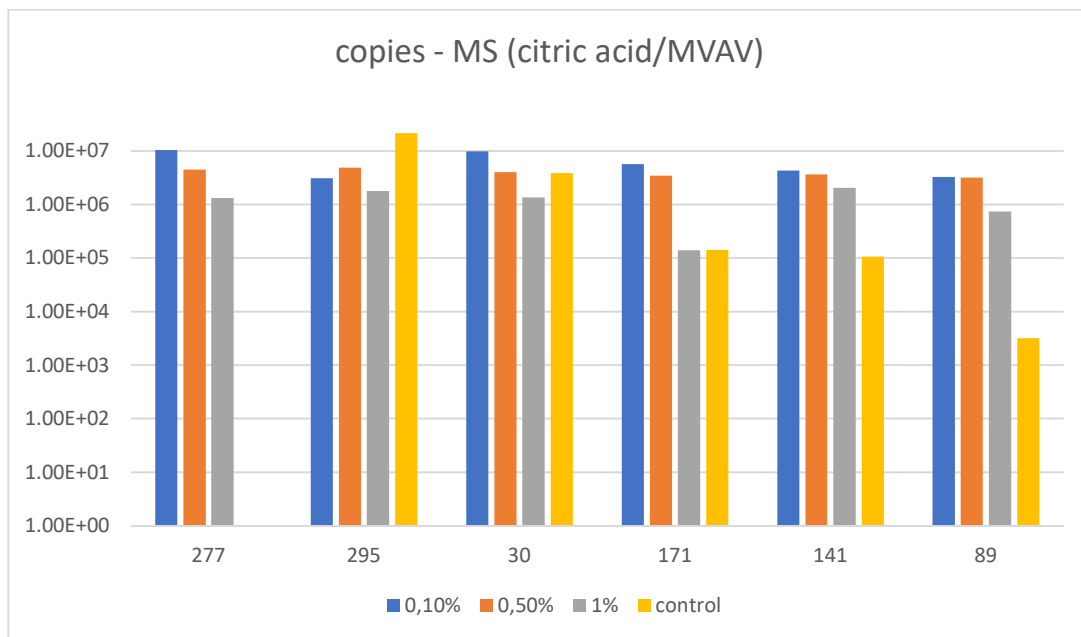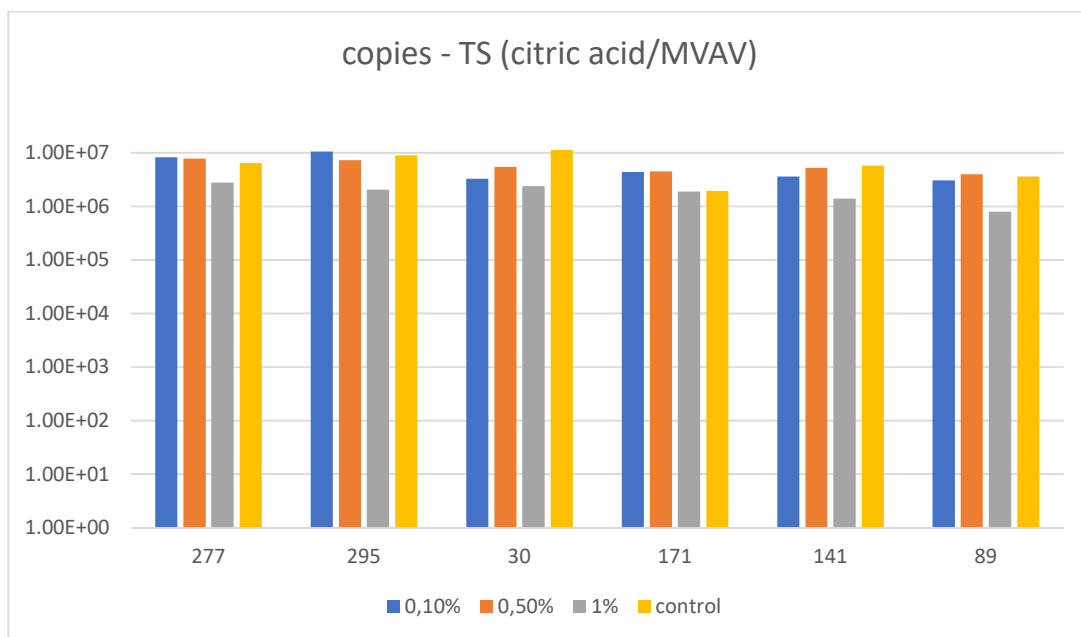

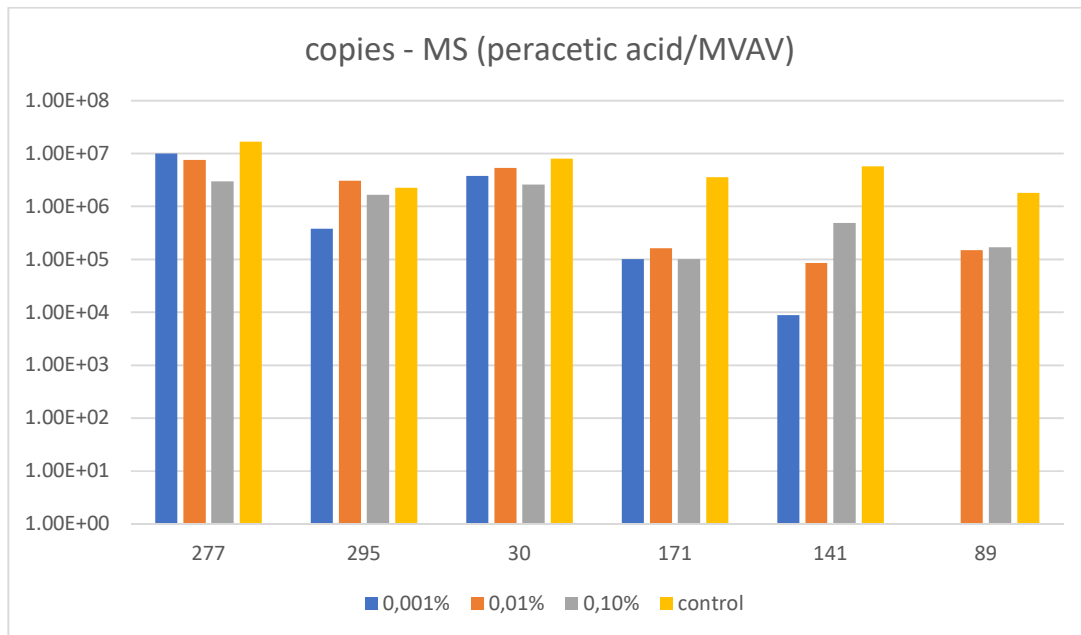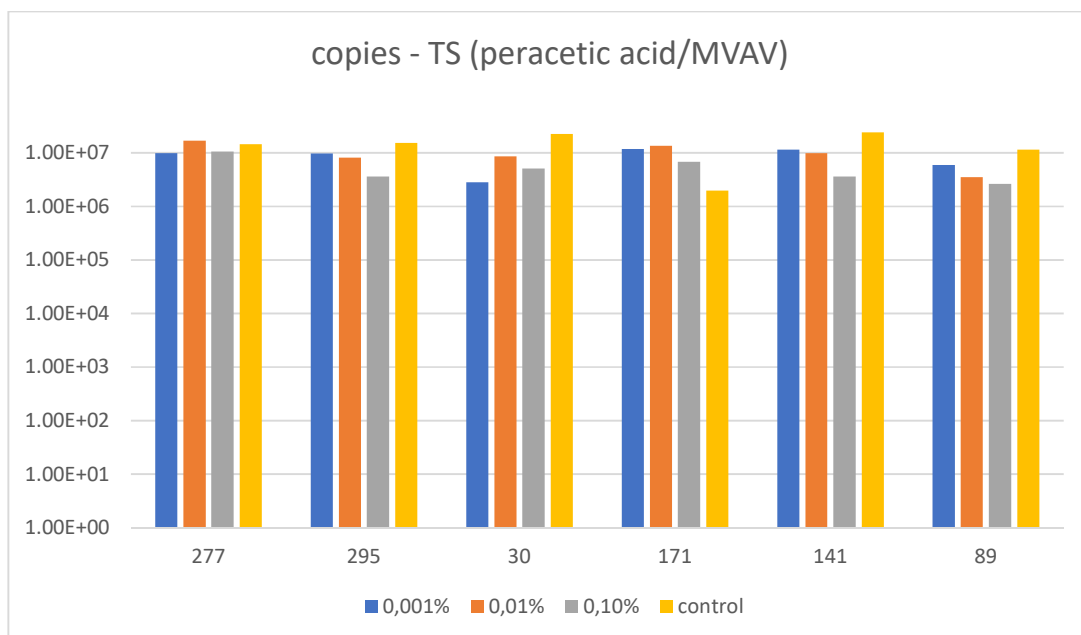

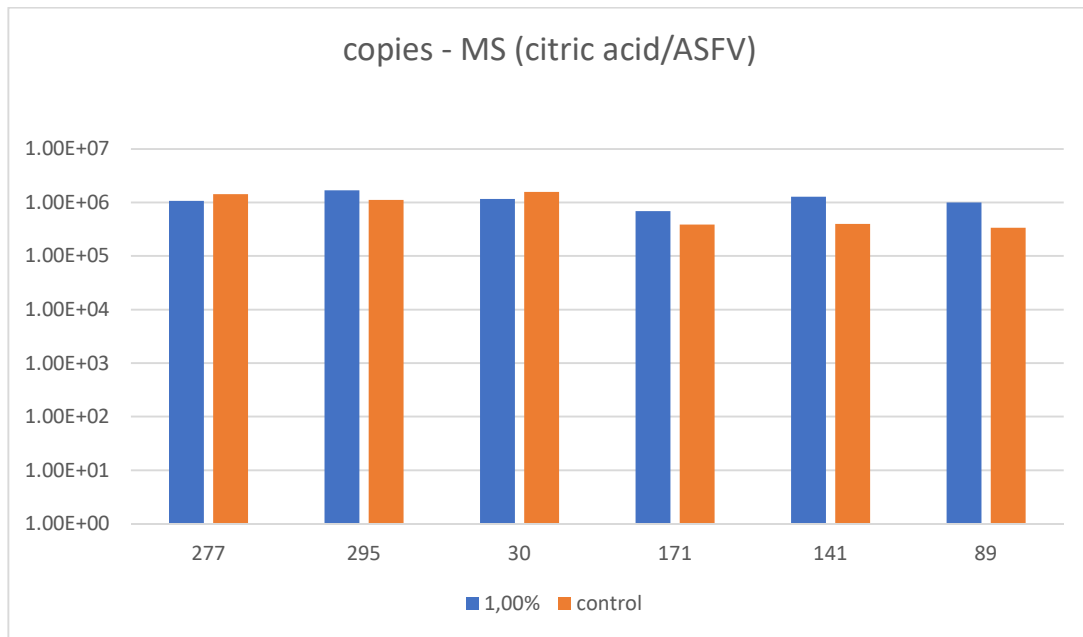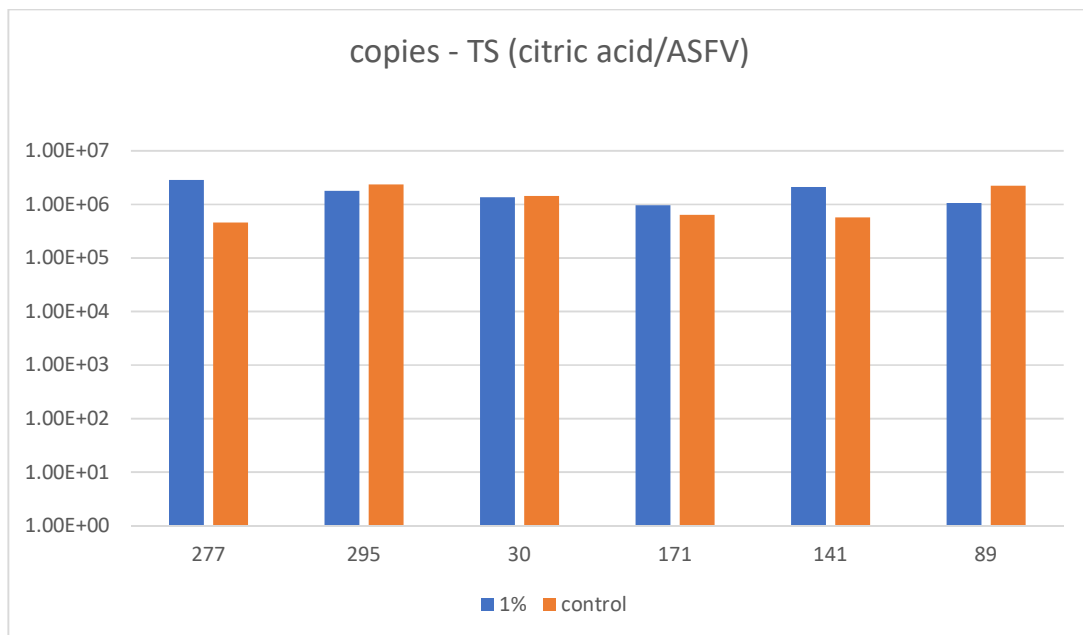

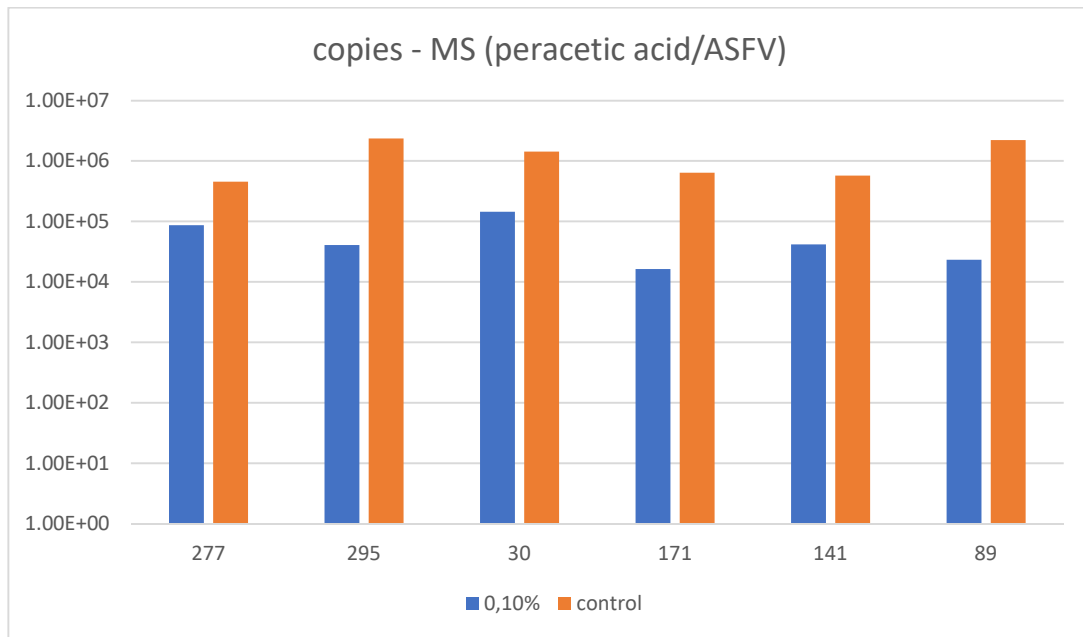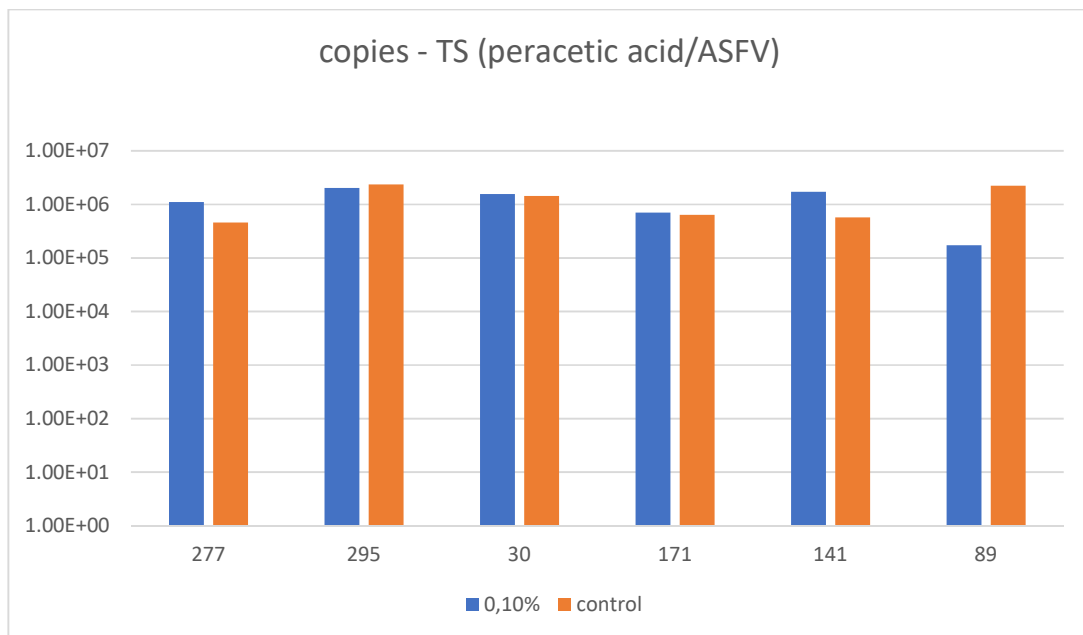

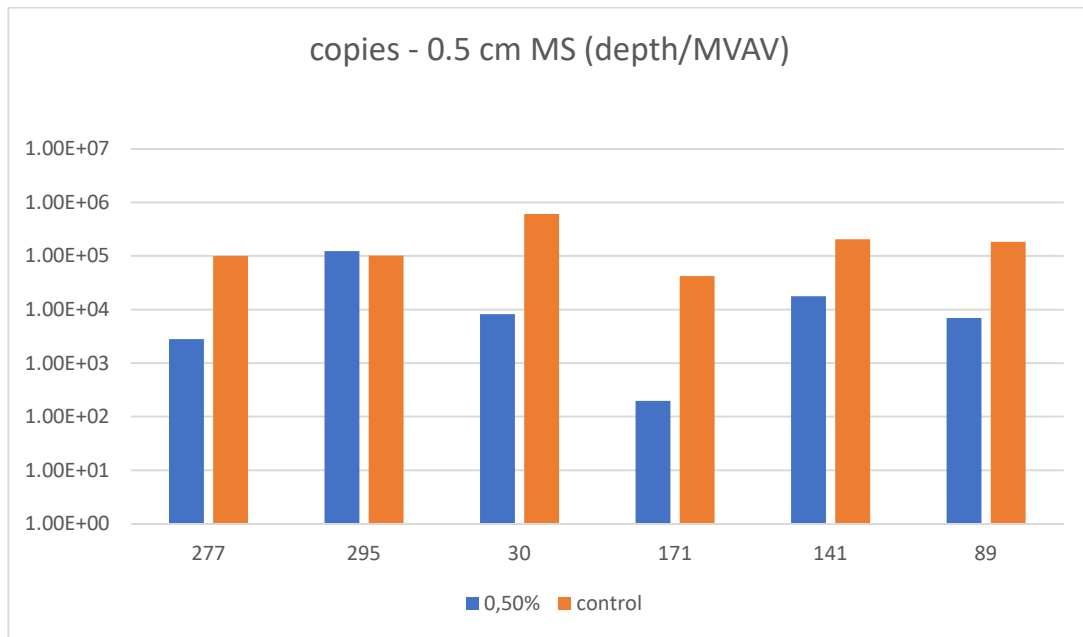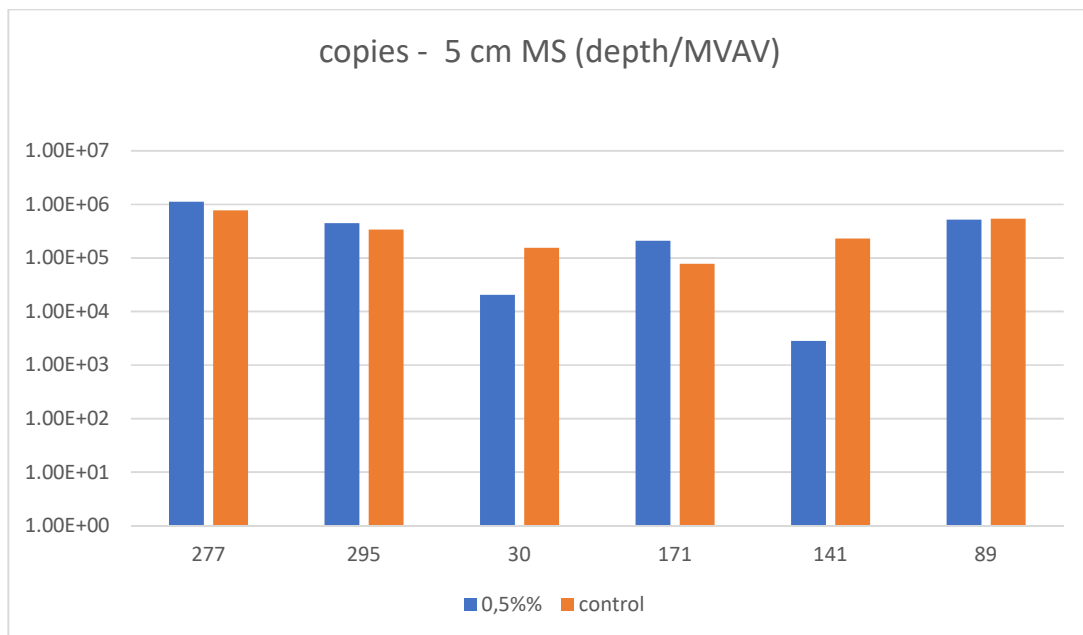

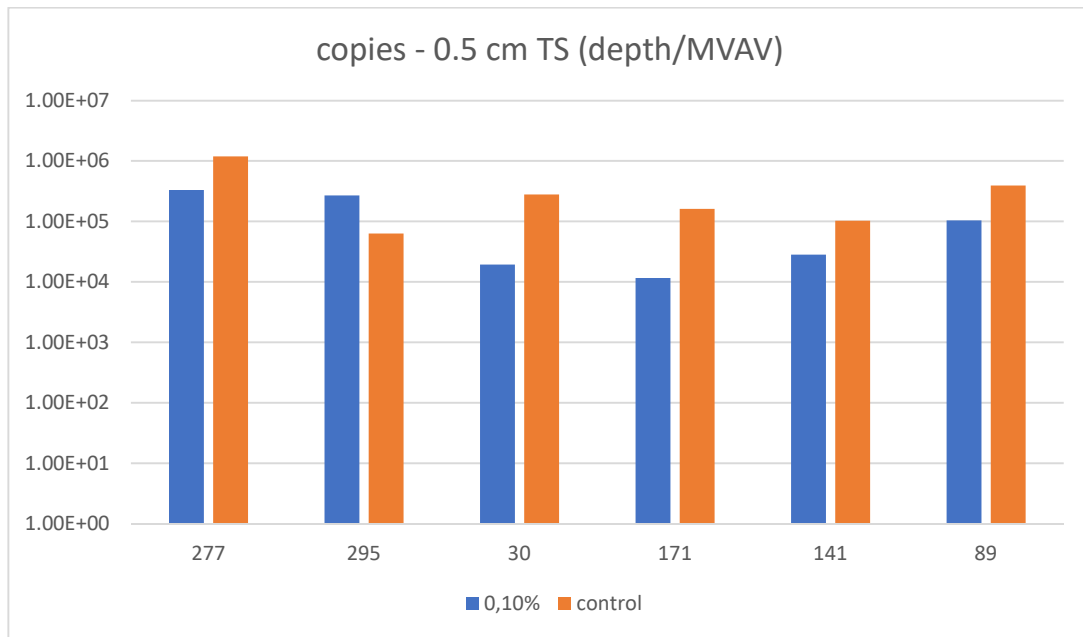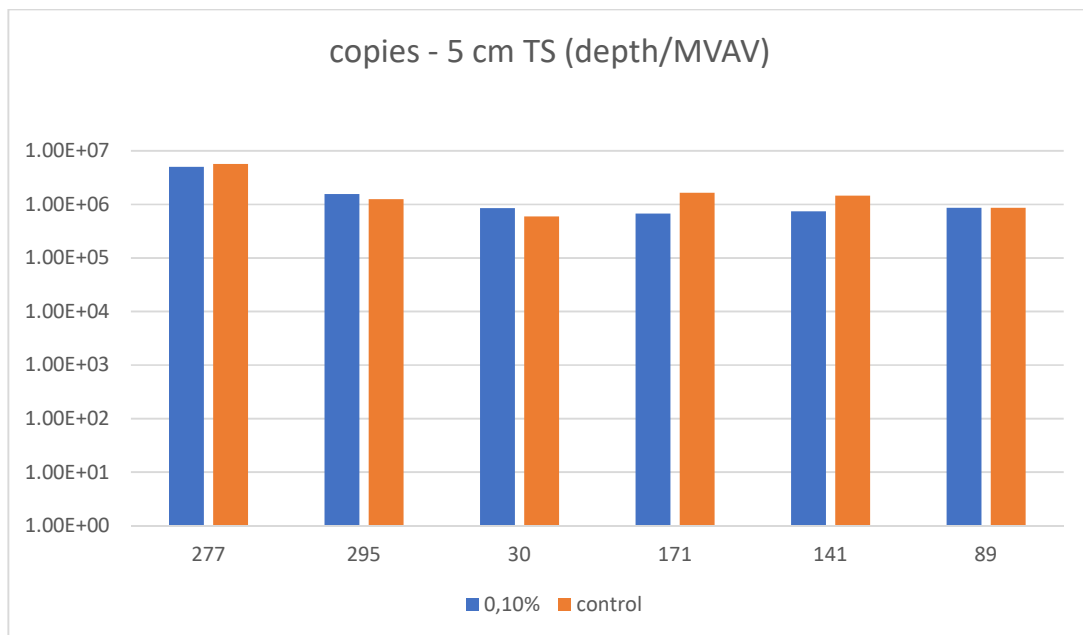

Supplement: Supplementary file 1 [file viruses-13-02173-s001.zip › viruses-1385496-supplementary.pdf]
